# Mixture of Phlorotannin and Fucoidan from *Ecklonia cava* Prevents the Aβ-Induced Cognitive Decline with Mitochondrial and Cholinergic Activation

**DOI:** 10.3390/md19080434

**Published:** 2021-07-29

**Authors:** Hye Ju Han, Seon Kyeong Park, Jin Yong Kang, Jong Min Kim, Seul Ki Yoo, Dae-Ok Kim, Gun-Hee Kim, Ho Jin Heo

**Affiliations:** 1 Division of Applied Life Science (BK21), Institute of Agriculture and Life Science, Gyeongsang National University, Jinju 52828, Korea; gksgpwn2527@korea.kr (H.J.H.); skp210316@kbri.re.kr (S.K.P.); kangjy2132@gnu.ac.kr (J.Y.K.); myrock201@gnu.ac.kr (J.M.K.); ysyk9412@gnu.ac.kr (S.K.Y.); 2 Animal and Plant Quarantine Agency, Gimcheon-si 39660, Korea; 3 Department of Neural Development and Disease, Korea Brain Research Institute (KBRI), Daegu 41062, Korea; 4 Department of Food Science and Biotechnology, Kyung Hee University, Yongin 17104, Korea; dokim05@khu.ac.kr; 5 Department of Food and Nutrition, Duksung Women’s University, Seoul 01369, Korea; ghkim@duksung.ac.kr

**Keywords:** *Ecklonia cava*, phlorotannin, fucoidan, amyloid-beta, cognition, mitochondrial function, tau phosphorylation, cholinergic function

## Abstract

The anti-amnesic effect of a mixture (4:6 = phlorotannin:fucoidan from *Ecklonia cava*, P4F6) was evaluated on amyloid-beta peptide (Aβ)-induced cognitive deficit mice. The cognitive function was examined by Y-maze, passive avoidance, and Morris water maze tests, and the intake of the mixture (P4F6) showed an ameliorating effect on Aβ-induced learning and memory impairment. After the behavioral tests, superoxide dismutase (SOD) activity and thiobarbituric acid-reactive substances (TBARS) contents were confirmed in brain tissue, and in the results, the mixture (P4F6) attenuated Aβ-induced oxidative stress. In addition, mitochondrial activity was evaluated by mitochondrial reactive oxygen species (ROS) content, mitochondrial membrane potential (MMP), adenosine triphosphate (ATP) content, and mitochondria-mediated apoptotic signaling pathway, and the mixture (P4F6) enhanced mitochondrial function. Furthermore, the mixture (P4F6) effectively regulated tau hyperphosphorylation by regulating the protein kinase B (Akt) pathway, and promoted brain-derived neurotrophic factor (BDNF) in brain tissue. Moreover, in the cholinergic system, the mixture (P4F6) ameliorated acetylcholine (ACh) content by regulating acetylcholinesterase (AChE) activity and choline acetyltransferase (ChAT) expression in brain tissue. Based on these results, we suggest that this mixture of phlorotannin and fucoidan (P4F6) might be a substance for improving cognitive function by effectively regulating cognition-related molecules.

## 1. Introduction

Alzheimer’s disease (AD) is the most common progressive neurodegenerative disease, and is characterized by cognitive decline [1,2]. Amyloid-beta (Aβ) is a critical factor involved in AD, namely, senile plaques and cerebral amyloid angiopathy [1]. Cognitive dysfunction is related to complex mechanisms (neuroinflammation, cholinergic system, amyloid plaque, nerve growth factor) [2]. At the center of the complex cognitive dysfunction-related signaling mechanism, the Aβ peptides play a central role [1]. Aβ peptide, which is generated by the enzymatic cleavage of APP by β-secretase and ϒ-secretase, is known as a neurotoxin. The formation of Aβ oligomers in synaptic terminals is correlated with cognitive dysfunction through neurotransmission inhibition and cell death [3,4,5]. The mechanism of Aβ toxicity is included in alteration of intracellular calcium and pro-apoptotic/inflammatory molecule production (nitric oxide, cytokines and reactive oxygen intermediates) [5,6]. Among the various mechanisms of cognitive decline, mitochondrial function is more emphasized, and is suggested as a treatment for neurodegenerative diseases such as AD and Parkinson’s disease (PD) [6]. Neurons are sensitive to bioenergetic fluctuations because they need a large amount of energy to perform neurotransmitter functions, and the synapses of neurons are enriched in mitochondria [3]. Mitochondria play a major role in programmed cell death (mitochondria-dependent pathway) by activating pro-apoptotic molecules in mammalian cells [3]. In addition, mitochondrial dysfunction can induce cholinergic dysfunction through the inhibition of ACh synthesis, and neurofibrillary tangles (NFTs) production by inactivation of tau protein kinase and glycogen synthase kinase (GSK)-3β [7]. Therefore, mitochondrial activation-targeting compounds are proposed as a major therapeutic strategy in neurodegenerative disease [6]. In particular, natural antioxidants are reported to play an important role in improving mitochondrial function [8].

*Ecklonia cava* (*E. cava*), edible brown algae, is used as a food ingredient, animal feed, fertilizer supplement, and herbal medicine based on its strong physiological activity [3]*. E. cava* are a rich source of bioactive compounds, mainly phlorotannins (phenolic compounds) and fucoidan (sulfated polysaccharide). Phlorotannins are formed by polymerizing phloroglucinol (1,3,5-tryhydroxybenzene) units as a basic structure through a polyketide pathway, and include eckol, bieckol, dieckol, and phlorofucofuroeckol [9,10,11]. The biological activities of phlorotannins are reported to include antioxidant, anti-tumor, and various enzyme inhibitory effects [9]. Most phlorotannins are reported to have a stronger antioxidant effect than commercial antioxidants such as α-tocopherol, butylated hydroxyl-anisole, and butylated hydroxytoluene [12]. Furthermore, the strong antioxidant activity is based at the center of the physiological activities of phlorotannins. The polysaccharide has energy storage functions and structural components in organisms, and the sulfated polysaccharides as cell wall components protect seaweed from tidal damage. In addition, fucoidan, mainly found in brown algae, consists of polymers formed by branched polysaccharide sulfate esters with L-fucose, and they include other monosaccharides such as mannose, galactose, glucose, and xylose [13]. Fucoidan has various structures and molecular weights from 43 to 1600 kDa, and is classified according to molecular weight and structure [14]. The specific bioactivity of fucoidan is determined by its unique structure. Among the various physiological activities, the major physiological activity of fucoidan is known to have anti-inflammatory and neuroprotective effects on ischemic reperfusion injury, and anti-coagulant and anti-thrombotic activity [13,15].

Cognitive decline is not caused by any simple mechanism, and the mechanism is still unclear. Although the ultimate goal of AD treatment is inhibition of Aβ plague, neurofibrillary tangles and synaptic failure, their signaling is closely linked to the regulation of oxidative stress and inflammatory response [16]. According to a recent study, a combination of natural antioxidants (carotenoid astaxanthin) and anti-inflammatory agents (omega-3 and omega-6 polyunsaturated fatty acids) has been proposed as a therapy for neurodegenerative disease [17]. In view of the report, a mixture of phlorotannins and fucoidan may have a more effective cognitive-enhancing effect than phlorotannin or fucoidan treatment alone. Furthermore, our previous findings revealed that the mixture (P4F6) was the optimal ratio for neuronal cell protection in human-derived MC-IXC cells and rat-derived PC-12 cells. The possibility of the mixture (P4F6) as therapeutic agent on TMT-induced cognitive dysfunction via mitochondrial activation and inhibitory effect of Aβ production/tau hyperphosphorylation was confirmed [18]. Therefore, for neurodegenerative disease, we evaluated the potential of the mixture (P4F6) from *E. cava* with the antioxidant and anti-inflammatory effects on Aβ-targeted cognitive dysfunction as a major AD hallmark.

## 2. Results

### 2.1. Behavioral Tests

**Figure 1:**
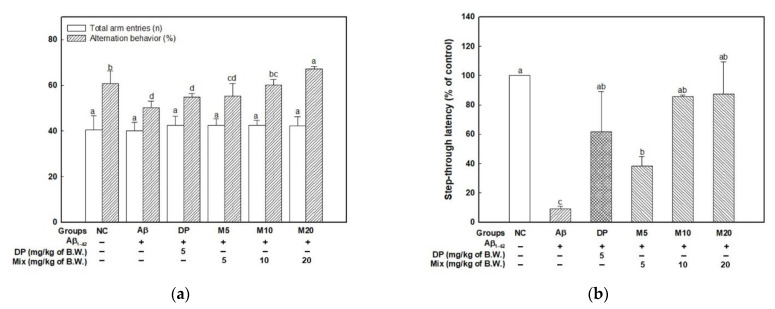

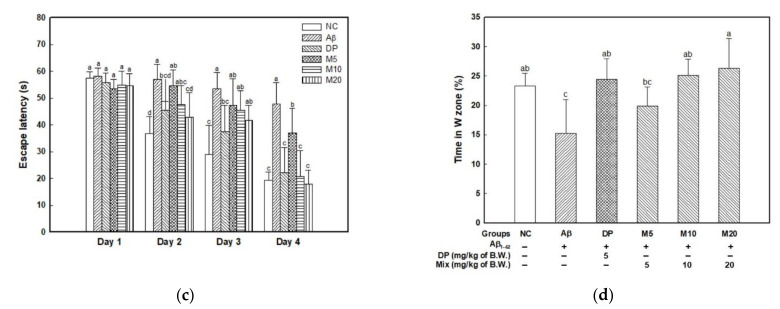
Ameliorating effect of mixture (4:6 = phlorotannin:fucoidan from *Ecklonia cava*, P4F6) on Aβ-induced learning and memory impairment. The spontaneous alternation behavior and number of arm entries (**a**) in the Y-maze test. Step-through latency time in the passive avoidance test (**b**). Escape latency in the hidden platform (**c**), time in the W zone for the probe trial (**d**) in the Morris water maze test. Values are mean ± SD (*n* = 9), and different small letters^(a–d)^ above the columns in the figures indicate statistical difference between each group (ANOVA and Duncan’s new multiple-range tests; *p* < 0.05).

To confirm the spatial learning and memory function, a Y-maze test was conducted (Figure 1a). The total arm entries showed similar movement in all groups. This means that the Aβ injection had no effect on motor ability. The Aβ group (50.31%) showed a significant reduction in spontaneous alternation behavior by approximately 10.55% compared with the NC group (60.86%) (Figure 1a). The alternation behavior of the mixture (P4F6) groups (M5; 55.35, M10; 60.11, and M20; 67.15%) was improved compared with the Aβ group, as well as the DP group (54.84%) used as a positive control, which was also improved.

Short-term learning and memory ability was confirmed using a passive avoidance test, and the results are shown in Figure 1b. The Aβ group (9.11%) saw a decrease in latency time compared with the NC group (100.00%). However, the mixture groups showed enhanced step-through latency (M5; 38.44, M10; 85.78, and M20; 87.44%) compared with the Aβ group.

Long-term learning and memory ability was measured with a Morris water maze test. During the hidden trial period to learn the location of the platform (days 1–4), the escape latency of all groups was gradually reduced (Figure 1c). On the last day of the hidden trial (day 4), the Aβ group spent a relatively long time escaping compared to the other group, suggesting that cognitive impairment due to Aβ had occurred. After the training trial, the results of the probe trial are shown in Figure 1d. The Aβ group (15.21%) decreased time in the W zone where the platform existed compared with the NC group (23.35%). In contrast, the DP group (24.44%), used as a positive control, ameliorated long-term learning and memory impairment by Aβ injection. In particular, the mixture groups (M10 and M20) also showed a similar improvement as the NC group with 25.15% and 26.34%, respectively.

### 2.2. Antioxidative Biochemical Analysis

**Figure 2:**
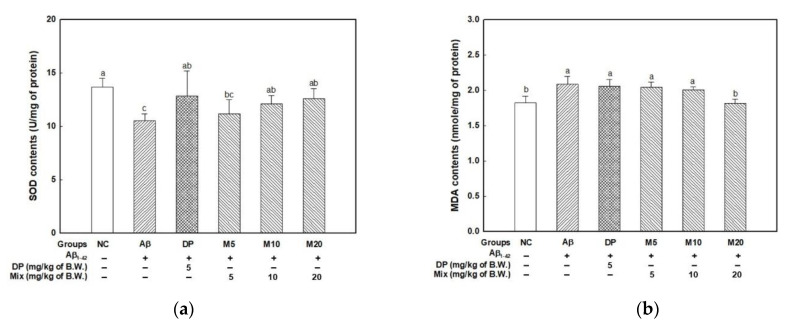
Antioxidant effect of mixture (4:6 = phlorotannin:fucoidan from *Ecklonia cava*, P4F6) on Aβ-induced oxidative stress. SOD (**a**) and TBARS (**b**) contents in brain tissue. Values are mean ± SD (*n* = 9), and different small letters (^a–c^) above the columns in the figures indicate statistical difference between each group (ANOVA and Duncan’s new multiple-range tests; *p* < 0.05).

To assess the antioxidative effect of the mixture (P4F6) in brain tissue, the SOD and TBARS content were measured (Figure 2). In Figure 2a, the Aβ group (10.54 U/mg of protein) indicated decreased SOD content compared with the NC group (13.65 U/mg of protein). On the other hand, the intake of DP (12.85 U/mg of protein) and mixture (M10; 12.11 and M20; 12.56 U/mg of protein) effectively stimulated antioxidant activity by increasing SOD content.

The TBARS contents were measured as a biomarker indicating the degree of lipid peroxidation (Figure 2b). The TBARS content of the Aβ group (2.08 nmole/mg of protein) increased by about 14.26% compared to the NC group (1.82 nmole/mg of protein). In addition, the mixture group (M20; 1.82 nmole/mg of protein) effectively inhibited lipid peroxidation.

### 2.3. Mitochondrial Activity

**Figure 3:**
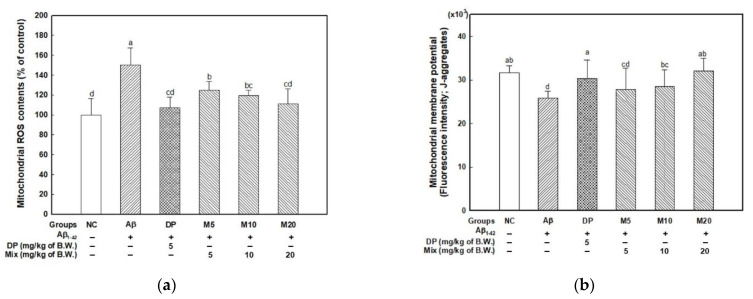

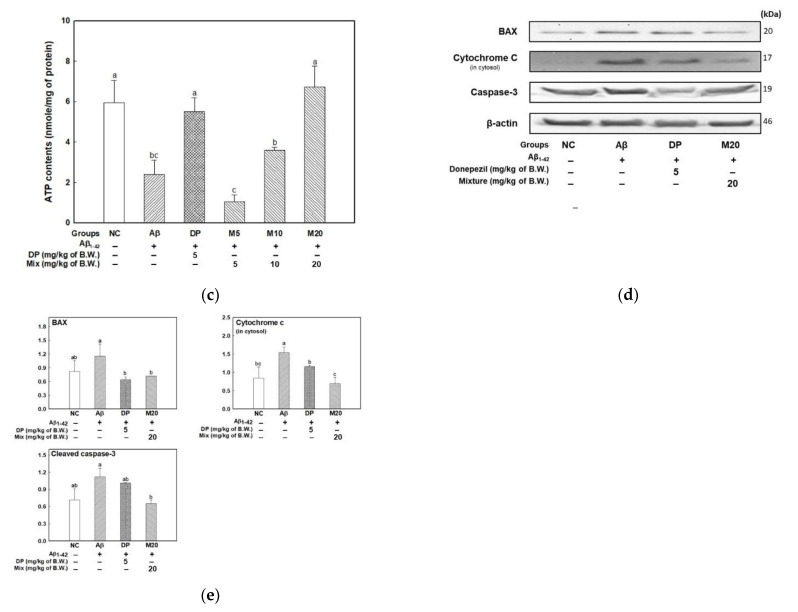
Mitochondrial activity of the mixture (4:6 = phlorotannin:fucoidan from *Ecklonia cava*, P4F6) on Aβ-induced mitochondrial damage. Mitochondrial ROS content (**a**), mitochondrial membrane potential (MMP) (**b**), and ATP content (**c**) on mitochondria isolated from brain tissue. Band images of Western blot analysis (**d**), the expression level of mitochondria-dependent apoptotic molecules (BAX, cytosolic cytochrome c, and caspase-3) (**e**) in mouse brain tissue. Values are mean ± SD (*n* = 5), and different small letters^(a–d)^ above the columns in the figures indicate statistical difference between each group (ANOVA and Duncan’s new multiple-range tests; *p *< 0.05).

To assess the ameliorating effect on Aβ-induced mitochondria damage, mitochondrial ROS content, MMP, ATP level, and the expression levels of mitochondria-mediated molecules were investigated (Figure 3). The mitochondrial ROS content increased with Aβ (150.25%) compared with the NC group (100.00%) (Figure 3a). In contrast, the DP (107.21%) and mixture groups (D5; 125.04, D10; 119.34, and D20; 110.88%) showed a significant inhibition of mitochondrial ROS production by Aβ injection.

In the MMP measurement results, the Aβ group (25,815.50 intensity; about 20.70 decrease) saw a decrease in MMP compared with the NC group (31,705.17 intensity), while the DP (30,338.50 intensity) and M20 (32,057.00 intensity) groups significantly restored MMP compared with the Aβ group (Figure 3b).

In Figure 3c, Aβ-induced mitochondria dysfunction led to a decrease in ATP level, and as a result, the Aβ group (2.40 nmole/mg of protein, about 59.73% decrease) showed decreased ATP content compared to the NC group (5.96 nmole/mg of protein). Meanwhile, the DP group (5.52 nmole/mg of protein) promoted ATP production. In addition, the M20 group (6.72 nmole/mg of protein) showed a significant increase in ATP level.

Based on the results, the intake of the mixture indicated the activation of mitochondria function by inhibiting mitochondrial ROS production and activating MMP and ATP production. Therefore, mitochondria-mediated apoptosis molecules were measured by analyzing BAX, cytochrome c (in cytosol), and caspase-3 (Figure 3d,e). The Aβ group induced BAX expression, and cytochrome c was released from mitochondria to cytosol. The cytochrome c released into the cytosol activated caspase-3. However, the administration of the mixture effectively counteracted the expression level of BAX and cytochrome c released into cytosol compared with the Aβ group. Finally, the expression level of caspase-3 was inhibited by the administration of the mixture (P4F6), and these results demonstrated that the mixture (P4F6) was effective material for mitochondrial activation through the down-regulating of mitochondria-mediated apoptosis molecules.

### 2.4. Cognitive Function-Related Mechanism

#### 2.4.1. Tau Hyperphosphorylation-Related Mechanisms

**Figure 4:**
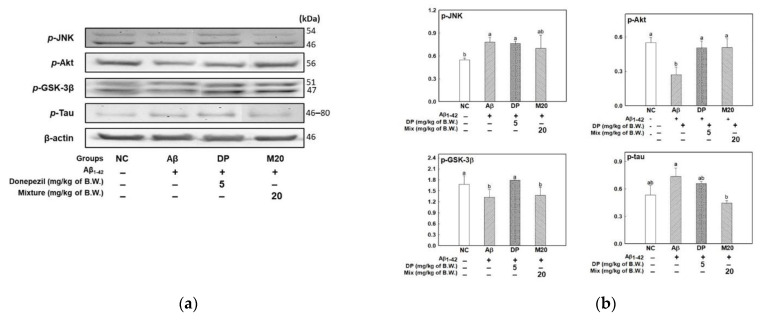
The regulating effect of the mixture (4:6 = phlorotannin:fucoidan from *Ecklonia cava*, P4F6) on Aβ-induced cognitive dysfunction. Band image of Western blot analysis (**a**), tau phosphorylation-related molecules (p-JNK, p-AKT, p-GSK-3β, and p-Tau) (**b**) in mouse brain tissue. Values are mean ± SD (*n* = 5), and different small letters (^a^^,b^) above the columns in the figures indicate statistical difference between each group (ANOVA and Duncan’s new multiple-range tests; *p* < 0.05).

The results of measurements of the Aβ-induced tau hyperphosphorylation signaling pathway are shown in Figure 4. The Aβ group was shown to have increased representation of p-JNK compared to the NC group, while the DP and M20 groups inhibited the expression of p-JNK (Figure 4a,b). The Aβ group showed decreased expression of p-Akt and p-GSK-3β, and eventually induced tau phosphorylation compared to the NC group. In contrast, the DP group was shown to inhibit tau hyperphosphorylation in brain tissue by promoting Akt phosphorylation, leading to GSK-3β inactivation (p-GSK-3β). The M20 group showed an increase in the expression of p-Akt, resulting in the inhibition of p-Tau.

#### 2.4.2. Cholinergic Function

**Figure 5:**
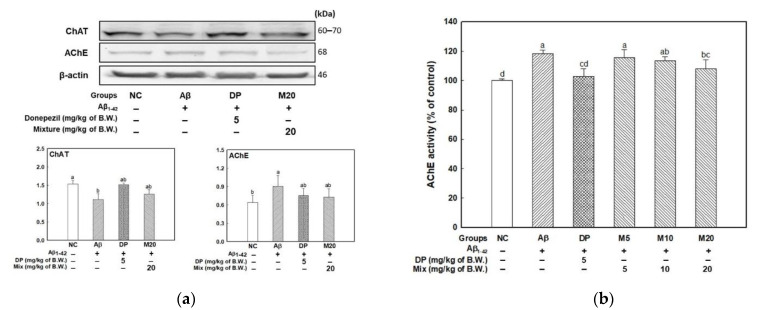

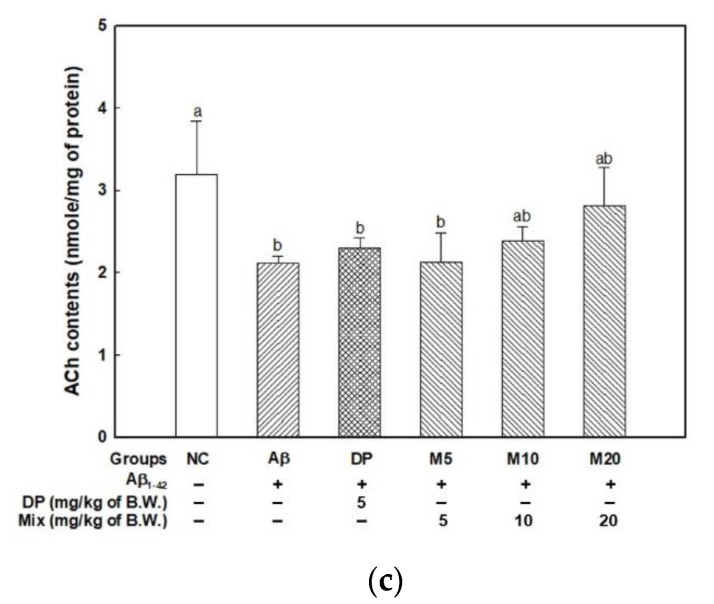
Effect of of the mixture (4:6 = phlorotannin:fucoidan from *Ecklonia cava*, P4F6) on Aβ-induced cholinergic dysfunction. AChE activity (**a**), band images and expression level of ChAT and AChE (**b**), and ACh contents (**c**) in mouse brain tissue. Values are mean ± SD (*n* = 9), and different small letters (^a–d^) above the columns in the figures indicate statistical difference between each group (ANOVA and Duncan’s new multiple-range tests; *p* < 0.05).

The cholinergic activity in brain tissue was evaluated by measuring ChAT and AChE expression levels, AChE activity, and ACh contents (Figure 5). The ChAT and AChE expression levels are presented in Figure 5a. The expression level of ChAT decreased and the expression level of AChE increased with the Aβ injection compared to the NC group. The M20 group showed a similar expression level compared with the DP group used as positive control. The ACh content is shown in Figure 5b, and the results showed that the ACh content of the Aβ group (2.11 nmole/mg of protein) increased by about 51.32% compared to that of the NC group (3.19 nmole/mg of protein). In contrast, that of the mixture groups (2.13, 2.39, and 2.82 nmole/mg of protein; M5, M10, and M20, respectively) showed a significant increase compared to the Aβ group. In addition, the activities of AChE are shown in Figure 5c. The Aβ group increased by about 118.41% compared to the NC group (100.00%), while the mixture groups (115.56%, 113.33%, and 107.94%; M5, M10, and M20, respectively) showed that inhibiting increased AChE activity.

## 3. Discussion

Aβ can cause cognitive deficits through complex mechanisms such as neuroinflammation, neuronal apoptosis, cholinergic dysfunction, and oxidative stress in the brain [19]. Recently, marine algae and their bioactive metabolites (phenolics, alkaloids, terpenoids, carotenoids, phytosterols, and polysaccharides) have attracted attention for the development of therapies for CNS diseases due to their neuroprotective effects [20]. Among the various bioactive compounds, phlorotannins and fucoidan were reported to be potential materials for cognitive function owing to their antioxidant, anti-inflammatory, and immunomodulatory capacities. The phlorotannin-rich extract from *E. cava* inhibited Aβ production and oligomerization in HEK293 cells and primary cell from rat cortical neurons of the embryonic brain [3]. Phlorotannin-rich extract from brown alga *Ishige foliacea* attenuated scopolamine-induced memory impairment with the regulation of cyclic AMP-response element-binding protein (CREB)-brain-derived neurotrophic factor (BDNF) expression [21]. The fucoidan isolated from *Sargassum fusiforme* (90 kDa) is being considered as a drug for AD patients due to its enhancing effect on scopolamine-, ethanol-, and sodium nitrite-induced cognitive deficits [22]. Moreover, fucoidan is also reported to have an inhibitory effect on Aβ accumulation within microglia [23]. In our results (Figure 1), the intake of the mixture (P4F6) effectively prevented Aβ-induced learning and memory impairment, suggesting that the mixture (P4F6) has the potential of being a preventative agent for neurodegenerative disease.

Aβ peptides are neurotoxins of the brain, and ROS generation, mitochondrial dysfunction and apoptosis are known as important mechanisms of neurotoxicity. In addition, Aβ oligomers induce serious oxidative stress by binding to hippocampal neurons [24]. ROS caused cell anomalies through enzyme deactivation, protein damage, DNA damage, and lipid peroxidation, and directly caused degradation of the cellular antioxidant system, which in turn leads to neurodegenerative diseases such as AD and PD [25,26]. In particular, the inhibition of SOD activity as major factor of the enzymatic antioxidant system accelerates mitochondria dysfunction through mtDNA damage and the release of cytochrome c resulting from the initiation of apoptosis [27]. Lipid peroxidation, occurring in neuronal membranes, is an indicator of the initiation, oxygenation, propagation, and termination of brain damage [28]. Phlorotannins including eckol, phlorofucofuroeckol A, dieckol, and 8,8’-bieckol are known as the strongest antioxidants. The radical scavenging activity of phlorotannins is determined by their structural characteristics with phenolic hydroxyl groups, and it is reported that there is a positive correlation between antioxidant effect and the number of hydroxyl groups [26]. In particular, the phenolic hydroxyl groups in the ortho position are known to act more efficiently on the inhibition of oxidative stress. Actually, phloroglucinol and eckol, which are composed of a basic structure, were detected in the brain. This means that they can pass through the B-B-B and be used as a direct antioxidant in brain tissue [29,30]. In addition, although fucoidan itself has no antioxidant activity, a previous study reported that fucoidan isolated from *Laminaria japonica* may affect endogenous antioxidants or oxidative stress through an increase in SOD, glutathione peroxidase (GSH-Px), and decrease in MDA content in the hippocampal tissue of Aβ-induced AD rat model [1]. Fucoidan also attenuated dopaminergic neuron death through potent antioxidative effects such as the inhibition of lipid peroxidation and activation of antioxidant enzyme (SOD, GSH-Px, and catalase) in a 1-methyl-4-phenyl-1, 2, 3, 6-tetrahydropyridine (MPTP)-induced Parkinson’s disease model [13]. Furthermore, SOD overexpression effectively ameliorated learning and memory function in a Tg2576 AD model [31]. Yuan and Macquarrie demonstrated that the antioxidant activity of fucoidan was associated with sulfate content and molecular weight [32]. However, the antioxidant mechanism of fucoidan is still unclear. Fucoidan extracted from *Fucus vesiculosus* (IC_50_ = 0.035 ± 0.002) exhibited a similar result in DPPH radical scavenging activity compared with quercetin (IC_50_ = 0.026 ± 0.001), a natural anti-oxidant [14]. In Figure 2, the administration of the mixture (P4F6) improved SOD activity and inhibited lipid peroxidation in Aβ-induced oxidative damage in brain tissue. Based on previous literature and our findings, the antioxidative effect of the mixture (P4F6) can be understood to be due to phlorotannin as an antioxidant itself and fucoidan as the activator of endogenous antioxidants against Aβ-induced oxidative stress in brain tissue. 

Mitochondrial dysfunction presents as a multisystem disease and has harmful effects on the CNS such as epilepsy, stroke-like episodes, ataxia, spasticity, and dementia [33]. Strong evidence suggests that mitochondrial damage and dysfunction occur early in neurodegenerative disease pathogenesis [34]. A recent study emphasized that mitochondrial function plays an important role in AD progression [29]. Mitochondria contribute to many cellular functions through the regulation of intracellular calcium ions (Ca^2+^), reduction-oxidation balance, and caspase-mediated apoptosis [30]. According to the mitochondrial cascade hypothesis, mitochondria trigger apoptosis by many stimuli. The mechanism of Aβ-induced mitochondrial structural and functional abnormalities in neuronal cells is still not clear. However, it was detected in AD patients that Aβ peptides induced the generation of free radicals in the mitochondrial membrane, and initiated mitochondrial dysfunction [35]. Aβ can be produced by mitochondria-associated APP metabolism, and enter into the mitochondrial matrix through the translocase of the outer/inner membranes [36]. Aβ and APP localized in mitochondrial membranes block the transport of nuclear-encoded mitochondrial proteins and collapse the electron-transport chain, resulting in mitochondrial dysfunction [29]. In addition, Aβ-induced oxidative stress affects the caspase-medicated apoptosis signaling pathway. Pro-apoptotic members such as Bax are translocated into the mitochondrial outer membrane from the cytoplasm. Bax can form pores, and cytochrome c is released into the cytoplasm [37]. The released cytochrome c binds to apoptotic activating factor-1 (Apaf-1), dATP, and caspase-9 to form a complex known as apoptosome. Apoptosome then activates caspase-9, and cleaves other caspase-3/7, leading to the apoptosis of neurons. Quercetin, which is a representative antioxidant of natural plants, inhibited senile plaque formation and ameliorated learning and memory impairment with the restoration of mitochondrial functions (e.g., MMP, ATP level and mitochondrial respiratory complexes) in the hippocampus of APPswe/PS1dE9 transgenic AD mice [8]. Resveratrol, a major phenolic compound of red wine, improved mitochondria function by inhibiting 3-nitrotyrosine and 4-hydroxynonenal and activating respiratory chain complexes in the immature brain during epileptogenesis [38]. Based on these reports, natural antioxidants are considered to be effective in improving mitochondrial function, and are proposed as a therapeutic agent for AD. According to Kang et al. (2013) [39], dieckol extracted from *Ecklonia cava*, a representative phlorotannin, effectively inhibits caspase-related apoptosis through ethanol-induced oxidative stress in a zebrafish model. In addition, fucoidan protected the cholinergic neurons in Aβ-induced neurotoxicity in primary neuronal cells from the rat basal forebrain by inhibiting ROS and caspase-3/9 [4]. Therefore, the mitochondrial activation of the mixture (P4F6) might be an optimal strategy for prevention on Aβ-induced cognitive dysfunction (Figure 3).

Tau is a microtubule binding molecule that maintains cytoskeletal stability, while tau hyperphosphorylation causes tau pathological aggregation (NFTs) and alteration of cytoskeletal stability, leading to subsequent loss of axonal transport and other cognitive signal-related functions [40]. NFTs as a major hallmark of AD could be formed by Aβ. Aβ in brain tissue activates tau protein kinase I and GSK-3β, resulting in tau phosphorylation [7], which is related to the regulation of many different kinases (GSK-3β, cyclin-dependent kinase 5, mitogen-activated protein kinases, protein kinase A/B/C/Novel, and calcium/calmodulin-dependent protein kinase II et al.) [41]. However, among many factors, the role of GSK-3 is reported to be important and is suggested as a therapeutic strategy for tau pathology. Aβ affects the phosphatidylinositol 3-kinase (PI3K)/Akt signaling pathway, which promotes cell survival and growth. p-PI3K activates p-Akt and p-GSK-3 (inactive form). By a Akt/GSK-3β-dependent mechanism, reduced p-Akt and p-GSK expression induce cell damage and death by causing tau phosphorylation in neurons [40]. According to Liu et al. [42], fucoidan from *Saccharina japonica* exhibited a neuroprotective effect on 1-methyl-4-phenyl pyridinium (MPP^+^)-induced cytotoxicity in SH-SY5Y cells through progressive activation of the PI3K/Akt signaling pathway. Fucoidan attenuated cell death in H_2_O_2_-induced oxidative damage in PC-12 cells, and the PI3K/Akt pathway played a key role in neuronal cell protection through the inhibition of Bax/Bcl-2 and caspase-3 activity [43]. According to a previous study, neuroglobin attenuated tau hyperphosphorylation by activating the Akt signaling pathway in Tg2576 (AD mice) and TgMapt (human tau over-expression mice) models, and it suggested a therapeutic target for AD [44]. In our study (Figure 4), the results provide evidence that the mixture (P4F6) has potential as an ameliorating material through the Aβ-induced tau hyperphosphorylation mechanism by activating the Akt signaling pathway.

Aβ and tau protein as a neurotoxin caused cholinergic dysfunction through the inhibition of ACh release and synthesis [45,46]. In particular, Aβ participated in allosteric modulation of the intrinsic catalytic efficiency of cholinesterases, resulting in synaptic and extrasynaptic cholinergic dysfunction [47]. ACh, which is a key neurotransmitter, is synthesized by ChAT. The action of ACh is terminated by AChE through the hydrolysis of ACh in synapses [1]. Therefore, ACh, AChE, and ChAT are used as cholinergic markers. The cholinergic system plays a critical role in cognitive function, and is affected by a variety of causes such as inflammation, oxidative stress, and mitochondrial dysfunction [1]. ACh synthesis is synthesized from acetyl-CoA and choline by ChAT in nerve terminals [5]. Aβ can inactivate pyruvate dehydrogenase, which converts pyruvate to acetyl-CoA in mitochondria, and cause a decrease in acetyl-CoA contents. Aβ also reduces the uptake of choline, which is transported into presynaptic neurons by membrane transport [5,7]. Finally, prolonged exposure and high doses of Aβ have been reported to inhibit ChAT activation [7]. Overall, Aβ affects the inhibition of ACh synthesis, and mitochondrial activation might be influenced by ACh synthesis and release. In addition, Aβ causes cholinergic cell damage and death through an excitotoxic pathway [2]. Aβ-AChE complex triggered the formation of amyloid fibrillogenesis and plaque through Aβ deposition and aggregation [2,48]. According to previous reports [49], treatment of the Aβ-AChE complex has been shown to further damage the neurite network in neurons more than treatment with Aβ alone. The phlorotannin-rich extract from *E. cava* including dieckol had an inhibitory effect against cholinesterases such as AChE and butyrylcholinesterase (BChE), and effectively protected PC-12 and SH-SY5Y cells in H_2_O_2_ and 2,2′-azobis(2-methylpropionamidine) dihydrochloride-induced cytotoxicity [50]. The sulfated polysaccharides from seaweed (*Ecklonia maxima*, *Gelidium pristoides*, *Ulva lactuca*, *Ulva rigida*, and *Gracilaria gracilis*) prevented Zn-induced oxidative damage of hippocampal neuronal (HT-22) cells with antioxidant molecules (SOD activity and glutathione contents) and inhibition of AChE activity [51]. In a recent study, it was described that Aβ, mitochondrial dysfunction and synaptic damage are linked to each other, and affect cognitive decline [29]. Recently, homotaurine, which inhibits the amyloidogenic pathway, demonstrated the activation of the cholinergic system via the regulation of γ-aminobutyric acid (GABA) A receptor affinity in mild cognitive impairment patients, and these findings suggest the possibility of drugs for AD patients [52]. Similar to previous literature, a mixture (P6F4) could be used as a potential agent for learning and memory impairment through cholinergic transmission activation with an anti-amyloidogenic effect. However, further studies conducted with properly designed experiments for animals and humans are necessary to fully demonstrate the action of the mixture (P4F6) for neurodegenerative disease.

Based on the report, the mixture (P4F6) was considered a potential material with mitochondrial activation, inhibitory effect of tau phosphorylation, and cholinergic activation in Aβ-induced cognitive decline. It is unclear whether there is a direct link with the targeted mechanism of the mixture among all these results (mitochondrial activation, inhibitory effect of tau phosphorylation, and cholinergic activation) or if this is a single phenomenon. However, our results considered that the mixture (P4F6) is a multi-targeted protective agent for Aβ-induced cholinergic dysfunction and mitochondrial damage.

## 4. Materials and Methods

### 4.1. Chemicals

Aβ_1-42_, acetylthiocholine, 5,5-dithiobis (2-nitrobenzoic acid) (DTNB), SOD assay kit, thiobarbituric acid (TBA), trichloroacetic acid (TCA), phenylmethanesulfonyl fluoride (PMSF), digitonin, egtazic acid (EGTA), and all the other chemicals used were obtained from Sigma-Aldrich Chemical Co. (St. Louis, MO, USA). Anti-p-tau (sc-12952), anti-cytochrome c (sc-13560), anti-p-Akt1/2/3 (sc-101629), anti-p-JNK (sc-6254), and anti-β-actin (sc-69879) were purchased from Santa Cruz Biotechnology (Santa Cruz, CA, USA). Secondary antibodies were purchased from Cell Signaling Technology (Danvers, MA, USA). Anti-ChAT (20747-1AP) was purchased from Proteintech (Wuhan, China). 

### 4.2. Sample Preparation

Phlorotannin extract from *E. cava* was extracted with 50% ethanol and filtered with a centrifuge, and the supernatant was collected. The extract contained dieckol (4.80%) as a major compound of *E. cava*. Fucoidan extract was extracted by acid hydrolysis, and filtered with a decanter. The supernatant was dried and used as fucoidan extract (Mw = 110.78 kDa, sulfate 3.03%, monosaccharides rate; arabinose (2.03%), fucose (15.19%), galactose (9.62%), glucose (11.42%), rhamnose (1.44%), xylose (23.77%), and other monosaccharides (36.53%)). The mixture of *E. cava* extract was mixed at a ratio of 4:6 (phlorotannin:fucoidan from *E. cava*) [18].

### 4.3. Animals

ICR mice (male, 4 weeks old) were purchased from Samtako (Osan, Korea), and the mice were housed in cages under controlled conditions (22 ± 2 °C, 50 ± 5 humidity, 12 h light/dark cycle). The mice were randomly divided into six groups (*n* = 15): a normal control (NC) group, an Aβ_1-42_-injected (Aβ) group (negative group), a donepezil 5 mg/kg of body weight (DP) group (positive group), and a mixture (P4F6) group (5, 10, and 20 mg/kg of body weight; M5, M10, and M20, respectively). The mixture (P4F6) and donepezil were dissolved in drinking water and orally fed using a stomach tube once a day for 4 weeks. After oral administration, Aβ_1-42_ was injected at the bregma (410 pmole, 10 μL in 1% NH_3_OH solution) using a 25 μL Hamilton microsyringe fitted with a 26-gauge needle that was inserted to a depth of 2.5 mm without anesthesia [53]. After injection, the mice were allowed to adapt for 3 days, and then the behavioral experiment was performed. Donepezil, an acetylcholinesterase inhibitor, is a drug approved for use in AD and other dementias, and alleviates the symptoms of AD. Therefore, in our study, we tried to confirm the effect of the mixture (P4F6) compared with donepezil used as a positive control on an Aβ-induced mice model [54].

### 4.4. Behavioral Tests

#### 4.4.1. Y-Maze Test 

The Y-maze consisted of three arms made of black acrylic plate with the same dimensions (33 cm long, 15 cm high and 10 cm wide), and each arm was set as A, B, and C. A mouse was located at the end of the arm, and its movement was recorded using a smart 3.0 video tracking system (Panlab, Barcelona, Spain) for 8 min [18].

#### 4.4.2. Passive Avoidance Test 

The passive avoidance test box consisted of a dark place that could give electrical stimulation and a bright place. The mice were placed in the bright place for 2 min, and then received an electronic shock (0.5 mA, 3 s) when they stepped in the dark place. The next day, the mice were placed in the bright place again and the step-through latency time was measured when they re-entered the dark place (maximum time limit: 300 s) [18].

#### 4.4.3. Morris Water Maze Test

A stainless-steel circular pool (150 cm in diameter and 60 cm in height) was divided into quadrants (E, W, N and S zones) and filled with squid ink (Cebesa, Valencia, Spain). In the center of the W zone, a black platform was located during the training periods. The mice were allowed to swim freely, and the escape latency time to the platform was recorded on video (maximum time: 60 s). Training trials were repeated four times a day for four days. Lastly, a probe trial was conducted without the platform for 90 s, and the time spent in the W zone was recorded [55].

### 4.5. Biochemical Analysis

#### 4.5.1. SOD Contents

After the behavioral experiment, the mice were sacrificed for biochemical studies, and the whole brain was dissected. Small pieces of brain tissue were homogenized with 10-fold volume of PBS, and the homogenized tissue was centrifuged at 400× *g* for 5 min, and the pellets obtained were extracted with five-fold volumes of 1× cell extraction buffer for 30 min. After extraction, centrifugation was performed at 10,000× *g* for 10 min, and the supernatant obtained was used in a SOD assay using a SOD determination kit (Sigma-Aldrich Chemical Co.).

#### 4.5.2. Levels of Thiobarbituric Acid-Reactive Substances (TBARS)

The homogenized tissue with PBS was centrifuged at 6000× *g* for 10 min to obtain the supernatants. The supernatants were mixed with 1% phosphoric acid and 0.67% TBA, and reacted at 95 °C for 1 h. After cooling, the MDA-TBA complex was measured at 532 nm using a spectrophotometer (UV-1201; Shimadzu, Kyoto, Japan).

### 4.6. Mitochondrial Activity

#### 4.6.1. Isolation of Mitochondria from Brain Tissue

To isolate the brain mitochondria, the whole brain tissue was added to five-fold volumes of isolation buffer (215 mM mannitol, 75 mM sucrose, 0.1% BSA, 20 mM HEPES (Na^+^), and 1 mM EGTA), and homogenated using a bullet blender (Next Advance Inc., Averill Park, NY, USA). The homogenate was spun down at 1300× *g* for 5 min, and the supernatant was centrifuged again at 13,000× *g* for 10 min. To remove the synaptosome, the pellet was mixed with isolation buffer containing 0.1% digitonin, and left on ice for 5 min. After that, the mixture was added to the isolation buffer and centrifuged at 13,000× *g* for 15 min. The pellet was re-mixed with the isolation buffer without 1 mM EGTA and centrifuged again at 10,000× *g* for 10 min. The last pellet was added to the isolation buffer without 1 mM EGTA to proceed with the experiment [8].

#### 4.6.2. Mitochondrial ROS Content

To assess the ROS content in the mitochondria, the isolated mitochondria were mixed with 25 μM DCF-DA in respiration buffer (125 mM KCl, 2 mM KH_2_PO_4_, 2.5 mM malate, 20 mM HEPES, 1 mM MgCl_2_, 5 mM pyruvate, and 500 μM EGTA) and left in a dark room for 20 min. After incubation, the fluorescence intensity was measured (excitation wave: 485 nm, emission wave: 535 nm) [8].

#### 4.6.3. Measurement of MMP

To measure MMP, assay buffer (5 mM malate and 5 mM pyruvate in the isolation buffer) and 1 μM JC-1 dye were added to the isolated mitochondria, and it was left in a dark room for 20 min [8]. The lipophilic JC-1 dye enters into heathy mitochondria, and forms red ‘J-aggregates’. Thus, the fluorescence intensity was measured using a fluorescence microplate reader (Infinite 200, Tecan Co., San Jose, CA, USA) at 485 nm (excitation wave) and at 590 nm (emission wave).

#### 4.6.4. ATP Contents

The ATP of the mitochondria was extracted with 1% TCA and 25 mM Tris-acetate buffer (pH 7.8). The extract was centrifuged at 10,000× *g* for 15 min, and the resulting supernatant was used in the experiment. The ATP level was measured using a commercial kit (Promega, Madison, WI, USA) with a luminescence meter (Promega, Madison, WI, USA).

### 4.7. Measurement of Protein Expression

The brain tissues of the experimental animals were homogenized using lysis buffer containing 50 mM Tris-HCl (pH 7.4), 150 mM sodium chloride, 0.25% sodium deoxycholate, 1 mM ethylenediaminetetraacetic acid, 1% NP40, 1 mM Na_3_VO_4_, 1 mM PMSF, and 1% protease inhibitors. After that, these homogenates were immediately centrifuged at 13,000× *g* at 4 °C for 10 min. The proteins were electrophoresed on sodium dodecyl sulfate polyacrylamide gel and transferred to a polyvinylidene difluoride membrane (Millipore, Billerica, MA, USA). The membranes were blocked with 5% skim milk solution to prevent the nonspecific binding of the other proteins, and then incubated with the primary antibodies overnight at 4 °C. After incubation, the membranes were reacted with the secondary antibodies for 1 h at room temperature. Western blot images were detected with a ChemiDoc imaging system (iBright Imager, Thermo-Fisher Scientific, Waltham, MA, USA).

### 4.8. Cholinergic Activity

#### 4.8.1. AChE Activity

The homogenated tissue with PBS was centrifuged at 13,000× *g* for 30 min at 4 °C. The supernatant was mixed with 50 mM sodium phosphate buffer (pH 8.0) and then incubated at 37 °C for 15 min. After incubation, the mixture was added to 500 μM substrate solution and reacted at 37 °C for 10 min. The absorbance was then measured at 405 nm [56]. 

#### 4.8.2. ACh Content

The supernatant was mixed with alkaline hydroxylamine reagent (2 M hydroxylamine in HCl and 3.5 N NaOH), and reacted at room temperature for 1 min. The mixture was added to 0.5 N HCl and 0.37 M FeCl_3_ in 0.1 N HCl, and measured at 540 nm using a microplate reader (Epoch 2; BioTek, Winooski, VT, USA) [57]. 

### 4.9. Statistical Analysis

All of the data were presented as means ± standard deviation (SD). The statistical significances between each group were calculated by one-way analysis of variance (ANOVA), and post hoc analysis was conducted using Duncan’s new multiple-range test (*p* < 0.05) with SAS software version 9.4 (SAS Institute, Cary, NC, USA). Different small letters in figures indicate statistical difference (*p* < 0.05).

## 5. Conclusions

This study tried to evaluate the preventive effect of a mixture (P4F6, phlorotannin:fucoidan from *E. cava* = 4:6) on Aβ-induced cognitive decline. The administration of the mixture (P4F6) protected against Aβ-induced learning and memory impairment. The mixture (P4F6) groups showed an anti-oxidative effect through SOD activation and decrease in TBARS contents in Aβ-induced oxidative stress in brain. The mixture (P4F6) also activated mitochondrial function, and effectively regulated mitochondria-dependence apoptosis molecules (Bax, cytochrome c, and caspase-3). Notably, the mixture (P4F6) effectively regulated tau hyperphosphorylation through the Akt signaling pathway. Finally, the mixture (P4F6) protected the cholinergic function in brain tissue through the inhibition of AChE activity and the increase in ChAT expression, resulting in the increase in ACh content on Aβ-induced cholinergic dysfunction. Based on these results, the mixture (P4F6) suggests the possibility of use as a material for prevention of Aβ-induced cognitive decline through the regulation of mitochondrial function, tau hyperphosphorylation, and cholinergic function. These multitargeted regulating results suggest that the mixture (P4F6) is a candidate neuroprotective agent for Aβ-induced cognitive dysfunction.
